# Neonatal health in Nepal: analysis of absolute and relative inequalities and impact of current efforts to reduce neonatal mortality

**DOI:** 10.1186/1471-2458-13-1239

**Published:** 2013-12-28

**Authors:** Deepak Paudel, Ishwar B Shrestha, Matthias Siebeck, Eva A Rehfuess

**Affiliations:** 1 Center for International Health, Ludwig Maximilians University, Munich, Germany; 2 United States Agency for International Development, Kathmandu, Nepal; 3 Department of Community Medicine and Family Health, Institute of Medicine, Tribhuvan University, Kathmandu, Nepal; 4 Institute for Medical Informatics, Biometry and Epidemiology, Ludwig Maximilians University, Munich, Germany

**Keywords:** Neonatal mortality, Rate ratio, Rate difference, Policy analysis, Developing country

## Abstract

**Background:**

Nepal has made substantial progress in reducing under-five mortality and is on track to achieve Millennium Development Goal 4, but advances in neonatal health are less encouraging. The objectives of this study were to assess relative and absolute inequalities in neonatal mortality over time, and to review experience with major programs to promote neonatal health.

**Methods:**

Using four nationally representative surveys conducted in 1996, 2001, 2006 and 2011, we calculated neonatal mortality rates for Nepal and for population groups based on child sex, geographical and socio-economic variables using a true cohort log probability approach. Inequalities based on different variables and years were assessed using rate differences (rd) and rate ratios (rr); time trends in neonatal mortality were measured using the annual rate of reduction. Through literature searches and expert consultation, information on Nepalese policies and programs implemented since 1990 and directly or indirectly attempting to reduce neonatal mortality was compiled. Data on timeline, coverage and effectiveness were extracted for major programs.

**Results:**

The annual rate of reduction for neonatal mortality between 1996 and 2011 (2.8 percent per annum) greatly lags behind the achievements in under-five and infant mortality, and varies across population groups. For the year 2011, stark absolute and relative inequalities in neonatal mortality exist in relation to wealth status (rd = 21.4, rr = 2.2); these are less pronounced for other measures of socio-economic status, child sex and urban–rural residence, ecological and development region. Among many efforts to promote child and maternal health, three established programs and two pilot programs emerged as particularly relevant to reducing neonatal mortality. While these were designed based on national and international evidence, information about coverage of different population groups and effectiveness is limited.

**Conclusion:**

Neonatal mortality varies greatly by socio-demographic variables. This study clearly shows that much remains to be achieved in terms of reducing neonatal mortality across different socio-economic, ethnic and geographical population groups in Nepal. In moving forward it will be important to scale up programs of proven effectiveness, conduct in-depth evaluation of promising new approaches, target unreached and hard-to-reach populations, and maximize use of financial and personnel resources through integration across programs.

## Background

### Insufficient progress in reducing neonatal mortality

While infant and under-five mortality rates in developing countries have declined significantly in the past decades, newborn mortality rates have decreased much more slowly [1]. An estimated 18% to 37% of neonatal deaths, defined as deaths occurring during the first 28 days of life, could be averted through expanded coverage of cost-effective interventions, if delivered through family or community approaches [2].

Nepal is one of the few developing countries considered “on track” to achieve Millennium Development Goal (MDG) 4 to reduce child mortality by two-thirds between 1990 and 2015, [3] with reductions in under-five mortality from 118 to 54 per 1,000 live births (54% reduction) and infant mortality from 79 to 46 per 1,000 live births (41% reduction) between 1996 and 2011 [4-7]. Over the same time period, the neonatal mortality rate decreased by only 34%, from 50 to 33 per 1,000 live births. Socio-economic disparities in neonatal mortality in Nepal have been well documented, for example, neonatal mortality is evidently higher among poor groups and socially disadvantaged castes [8-10]. These are likely to be among the reasons for insufficient progress made to date, as interventions rarely reach all population groups in equitable ways.

### Direct and underlying causes of neonatal deaths

Globally, the main direct causes of neonatal death are preterm birth (28%), severe infections (26%), asphyxia (23%), and neonatal tetanus (7%) [11]. Information about the causes of neonatal deaths is limited in Nepal. Verbal autopsy findings of newborn deaths in the 2006 Nepal Demographic and Health Survey showed that major causes of death are infections (39%), birth asphyxia/birth injury (33%), congenital anomalies (8%) and pre-maturity or low birth weight (6%) [5]. Other Nepalese community- and hospital-based data also suggest infections, birth asphyxia, preterm birth and hypothermia as the most important causes, [12-14] largely in agreement with the general picture of the developing world.

In addition to lack of basic prenatal, natal and postnatal healthcare, a range of socio-economic and cultural factors, such as inability to pay for transportation and services, poor knowledge and attitudes in relation to healthcare, and various forms of gender bias negatively affect newborn survival in developing countries [15]. For example, in most of the cultures in rural Nepal, mothers are considered ritually “polluted” until *nwaran* (the name-giving ceremony on the ninth or eleventh day) and are restricted to stay at home, preventing access to care during this critical period. Also, newborn bathing immediately after birth and applying oil and turmeric powder to the cord stump are commonly practiced traditions and are known to increase the risk of neonatal infections [16].

### Community-level efforts to improve prevention and care

Critical interventions to reduce neonatal mortality include behavior change communication; community mobilization and engagement for improved antenatal, intrapartum, and postnatal care practices; and community-based case management of illness [2]. A major challenge in relation to these is promoting demand for healthcare and meeting this demand through interventions delivered at family and community levels. Indeed, studies from South Asia demonstrate that simple community- and home-based prevention and treatment interventions during pregnancy, birth and the post-natal period can effectively save the lives of newborns [17-19].

Drawing on international, regional and national evidence, the Government of Nepal initiated a series of policies and programs to address neonatal mortality in Nepal with efforts delivered through the governmental health system and its hospitals and peripheral health facilities (i.e. primary health care centres, health posts and sub-health posts) as well as through the strong workforce of 48,000 female community health volunteers. To date, no comprehensive review and analysis of these policies and programs has been undertaken with respect to their implementation and impact on newborn health.

The objectives of this study are to assess relative and absolute socio-economic inequalities in neonatal mortality over time, and to review current experience with programs to promote neonatal health in relation to progress towards achieving MDG 4.

## Methods

With respect to the first objective, the study used data from national surveys conducted in 1996 (Nepal Family Health Survey, NFHS) and 2001, 2006 and 2011 (Nepal Demographic and Health Surveys, NDHS). These surveys provide nationally representative data on fertility, health care behaviour and practices, childhood mortality, nutrition, and knowledge of HIV/AIDS that are comparable across different countries and across time. Data are in the public domain and accessible from the MEASURE DHS website (http://www.measuredhs.com). The surveys are based on two-stage, systematic cluster random sampling, and are characterized by response rates above 90%. Trained enumerators collect information from households and respondents after obtaining verbal informed consent. Table 1 shows the sample size and response rate for each survey. More details on the sampling methodology are available separately [4-7]. These surveys were reviewed and approved by the Institutional Review Board of the Nepal Health Research Council, Nepal; interviews were conducted after informed consent and the datasets used for this analysis were anonymous.

**Table 1:** Number of households, women of reproductive age and births by survey year

	**NFHS 1996**	**NDHS 2001**	**NDHS 2006**	**NDHS 2011**
Total households	8,082	8,602	8,707	10,826
*Response rate (%)*	*99.6*	*99.6*	*99.6*	*99.4*
Total women aged 15–49 years	8,429	8,726	10,793	12,674
*Response rate (%)*	*98.2*	*98.2*	*98.4*	*98.1*
Total births in last ten years	14,259	14,044	11,531	11,225
Approximate timeframe covered	1986-1995	1991-2000	1996-2005	2001-2010

This paper assesses time trends in neonatal mortality, which is defined as the number of deaths per 1,000 live births occurring during the first 28 days of life. We determined neonatal mortality rate based on a true cohort log probability approach [20] for babies born during the 10 years preceding the survey. In addition to calculating national averages, we disaggregated neonatal mortality by child sex, place of residence (i.e. urban, rural), ecological zone (i.e. mountain, hill, terai or flatland), development region (i.e. Eastern, Central, Western, Mid-Western, Far-Western), maternal education (i.e. no education, primary education, secondary or higher education), wealth quintile, and caste and ethnicity. To assess magnitude and trends in inequalities, we calculated rate differences (highest – lowest) as absolute measures of inequality and rate ratios (highest/lowest) as relative measures of inequality for each of the four survey periods. The rate ratio is unit-less and independent of average levels and scale, whereas the rate difference depends on both average levels and scale [21-23]. These two commonly used measures of inequality are easy to understand, but comparisons are limited to two extreme groups rather than covering the full population spectrum [24]. Reporting both absolute and relative measures of inequality is recommended to increase transparency, reduce systematic reporting biases, and improve the evidence base for policies aimed at reducing health inequalities [25].

The annual rate of change is commonly used to describe trends in increment (e.g. improved coverage) or reduction (e.g. reduced mortality rate), and to make projections of rates into the future. The annual rate of reduction (ARR) in neonatal mortality for this study was calculated as

$$\text{ARR}=\frac{\text{LN}\left({\text{NMR}}_{t1}/{\text{NMR}}_{t0}\right)\times 100}{\left(t1‒t0\right)}$$

where LN is the natural logarithm, NMR is the neonatal mortality rate, and t0 and t1 correspond to 1996 and 2011 respectively [26]. Analyses were conducted in Stata Special Edition version 12 [27].

With respect to the second objective, the study compiled information on all policies and programs implemented since 1990 that have directly or indirectly attempted to reduce neonatal mortality. We conducted a range of searches in the peer-reviewed literature, using PubMed, and in the grey literature, using the websites and electronic repositories of the Nepal Ministry of Health and Population (e.g. http://www.mohp.gov.np, http://www.dohs.gov.np, http://elibrary-mohp.gov.np) and of key donors such as the US Agency for International Development (e.g. http://dec.usaid.gov) and the UK Department for International Development (e.g. http://www.dfid.gov.uk/r4d), as well as through direct contact with individuals in these and other organizations. Relevant documents identified included scientific publications, annual reports, project reports and technical briefs. These were reviewed to identify existing policies and programs and to select major policies and programs to improve newborn health. For the latter, information was extracted to provide a brief description of activities and to document program timeline, scale and coverage, as well as program effectiveness.

## Results

### Time trends and socio-economic inequalities in neonatal mortality

**Figure 1:**
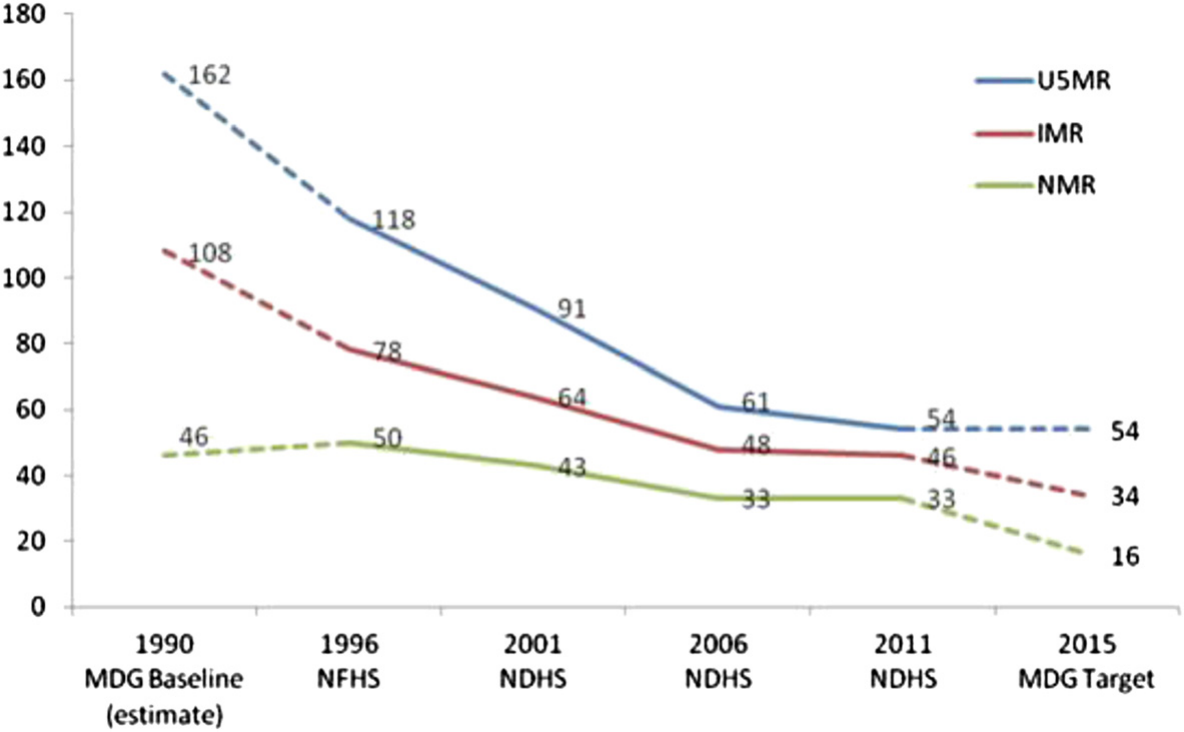
**Trend in child, infant and neonatal mortality in Nepal for 1990 to 2011 in relation to the MDG baseline for 1990 and MDG targets for 2015.** Note: Estimates of child, infant and neonatal mortality are based on the five-year period preceding the surveys. The MDG baseline is not survey-based but was estimated based on backward extrapolation of trends. Neonatal mortality does not form part of the MDG indicators, and the values for MDG baseline and MDG target are taken from the Nepali national health plan. U5MR: Under five mortality rate; IMR: Infant mortality rate; NMR: Neonatal mortality rate; MDG: Millenium Development Goal; NFHS: Nepal Family Health Survey; NDHS: Nepal Demographic and Health Survey.

The most recent estimates for neonatal, infant and under-five mortality in Nepal are 33, 46 and 54 per 1,000 live births respectively, for the period 2006-2011 [6]. The overall rate of reduction in childhood mortality between 1990 and 2011 is impressive; however, there are stark differences in the annual rate of reduction for under-five, infant and neonatal mortality (5.2, 3.6 and 2.8 percent per annum respectively for the five-year period preceding the survey). As shown in Figure 1, the country had already achieved the MDG 4 target for under-five mortality by 2011, but reductions in infant and neonatal mortality are a must if childhood survival is to improve further.

Table 2 shows inequalities in newborn mortality by child sex, geographical location (as measured by urban–rural residence, ecological region and development region) and socio-economic status (as measured by maternal education, wealth status and caste and ethnicity); these inequalities are evident in all four surveys. Male neonates from rural areas, living in mountain areas and the Far-Western region, born to mothers with no education and into families belonging to the lower wealth quintile and to a marginalized caste or ethnic group (*Dalit and Janajati)* are dying more frequently than their counterparts.

**Table 2:** Neonatal mortality rate for the 10-year period preceding the survey, by child sex, geographical location and socio-economic characteristics*

	***Neonatal mortality rate***				***Annual rate of reduction***
	**NFHS 1996**	**NDHS 2001**	**NDHS 2006**	**NDHS 2011**	***(1996–2011)***
Child sex					
Male	65.1	51.8	38.6	36.9	3.8
Female	49.6	42.6	36.8	33.1	2.7
*Rate difference*	*15.5*	*9.2*	*1.8*	*3.8*	*na*
*Rate ratio*	*1.3*	*1.2*	*1.0*	*1.1*	*na*
Residence					
Urban	43.2	35.9	24.6	25.3	3.6
Rural	58.5	48.1	39.6	36.2	3.2
*Rate difference*	*15.3*	*12.2*	*15.0*	*10.9*	*na*
*Rate ratio*	*1.4*	*1.3*	*1.6*	*1.4*	*na*
Ecological region					
Mountain	70.8	63.7	58.9	45.6	2.9
Hill	50.3	41.9	28.6	32.9	2.8
Terai	61.7	49	41.4	35.1	3.8
*Rate difference*	*20.5*	*21.8*	*30.3*	*12.7*	*na*
*Rate ratio*	*1.4*	*1.5*	*2.1*	*1.4*	*na*
Development region					
Eastern	56.7	50.1	32.5	29.3	4.4
Central	55.5	47.6	34.8	36.7	2.8
Western	52.0	38.9	34.5	37.0	2.3
Mid-western	63.0	40.3	55.9	33.6	4.2
Far-western	67.0	63.8	39.7	40.9	3.3
*Rate difference*	*15.0*	*24.9*	*23.4*	*11.6*	*na*
*Rate ratio*	*1.3*	*1.6*	*1.7*	*1.4*	*na*
Maternal education					
No education	59.5	51.1	43.3	40.3	2.6
Primary	51.6	41.1	34.1	33.6	2.9
Secondary or higher	41.6	24.3	20.3	26.2	3.1
*Rate difference*	*17.9*	*26.8*	*23.0*	*14.1*	*na*
*Rate ratio*	*1.4*	*2.1*	*2.1*	*1.5*	*na*
Wealth status					
Poorest	56.4	48.5	42.7	35.6	3.1
Poorer	63.4	56.0	37.6	40.0	3.1
Middle	65.8	46.9	46.9	39.2	3.5
Richer	53.3	47.2	30.4	36.9	2.5
Richest	47.0	32.1	26.3	18.6	6.2
*Rate difference*	*18.8*	*23.9*	*20.6*	*21.4*	*na*
*Rate ratio*	*1.4*	*1.7*	*1.8*	*2.2*	*na*
Caste and ethnicity					
Brahmin, Chhetri, Newar	52.6	43.9	33.1	31.0	3.5
Dalits	58.1	51.6	43.9	36.4	3.1
Janajati	51.7	47.9	34.0	34.6	2.7
Other	72.2	49.2	44.5	42.6	3.5
*Rate difference*	*20.5*	*7.7*	*11.4*	*11.6*	*na*
*Rate ratio*	*1.4*	*1.2*	*1.3*	*1.4*	*na*
**National**	**57.5**	**47.2**	**37.7**	**35.1**	**3.3**

*In each survey, the groups with the highest and lowest neonatal mortality were used to calculate rate differences and rate ratios. Please note some fluctuation between years in terms of the groups performing best or worst.

#### Annual rate of reduction

The overall average annual rate of reduction in neonatal mortality for the period 1996 to 2011 is 3.3 percent per year. The rate of reduction is greatest for the richest wealth quintile (6.2 percent per annum), and is also substantially above average for the Eastern development region (4.4 percent per annum) and the Mid-Western development region (4.2 percent per annum). Neonates living in the Western development region (2.3 percent per annum), or born into the richer wealth quintile (2.5 percent per annum), into a *Janajati* family (2.7 percent per annum) or to mothers with no education (2.6 percent per annum) show particularly low average annual rates of reduction.

#### Absolute inequalities based on rate differences in 2011

In Nepal, differences in neonatal mortality are most pronounced for wealth (21.4 between the wealth quintiles with highest and lowest neonatal mortality rates). Interestingly, neonatal mortality is higher among poorer and middle quintile families than among poorest quintile families. Differences in neonatal mortality rate are also relatively stark for maternal education (14.1 between a child born to a mother with secondary or higher education and a child born to a mother with no education). Differences are moderate for the three geographical indicators (10.9 for urban compared to rural areas, 12.7 for mountain compared to hill areas, 11.6 for the Far-Western compared to Eastern region), as well as caste and ethnicity (11.6 for Brahmins, Chhetris and Newars compared to others; others include diverse castes and ethnic groups that could not be disaggregated due to small sample sizes). Interestingly, absolute differences in neonatal mortality are not very pronounced for males compared to females (3.8).

#### Relative inequalities based on rate ratios in 2011

Overall, relative inequalities in neonatal mortality show similar results, with wealth status showing the greatest inequalities (2.2 for the richest wealth quintile compared to the poorer wealth quintile). Maternal education (1.5 for children born to mothers with no education compared to children born to mothers with secondary or higher education), geographical features (1.4 for urban–rural, Far-Western compared to Eastern and mountain compared to hill regions) and caste (1.4 for Brahmins, Chhetris and Newars compared to others) show very similar relative inequalities. Relative inequalities are barely present for male versus female neonates.

#### Changes over time

No clear and consistent pattern emerges in the reduction of absolute and relative inequalities in neonatal mortality based on the range of variables assessed. For most variables, rate differences and rate ratios are relatively stable with some fluctuation (i.e. urban–rural residence, development region, caste and ethnicity) or stark fluctuation (i.e. ecological region, maternal education) between years. For most comparisons, the groups with the highest and lowest neonatal mortality rates remain the same across comparisons; with caste and ethnicity, there is substantial variation between years. Findings for child sex and wealth status stand out: For child sex, rate differences and rate ratios were much more pronounced in 1996 and have shown a steady decline since then. No clear gradient for neonatal mortality emerges across wealth quintiles; the richest wealth quintile performs best across all four surveys but the worst performance is observed for either the poorer or middle wealth quintile rather than the poorest. Overall, rate differences are relatively stable over time whereas the rate ratio increased from 1.4 in 1996 to 2.2 in 2011 between wealth quintiles with the highest and lowest mortality rates.

### Major policies and programs to improve neonatal health

**Figure 2:**
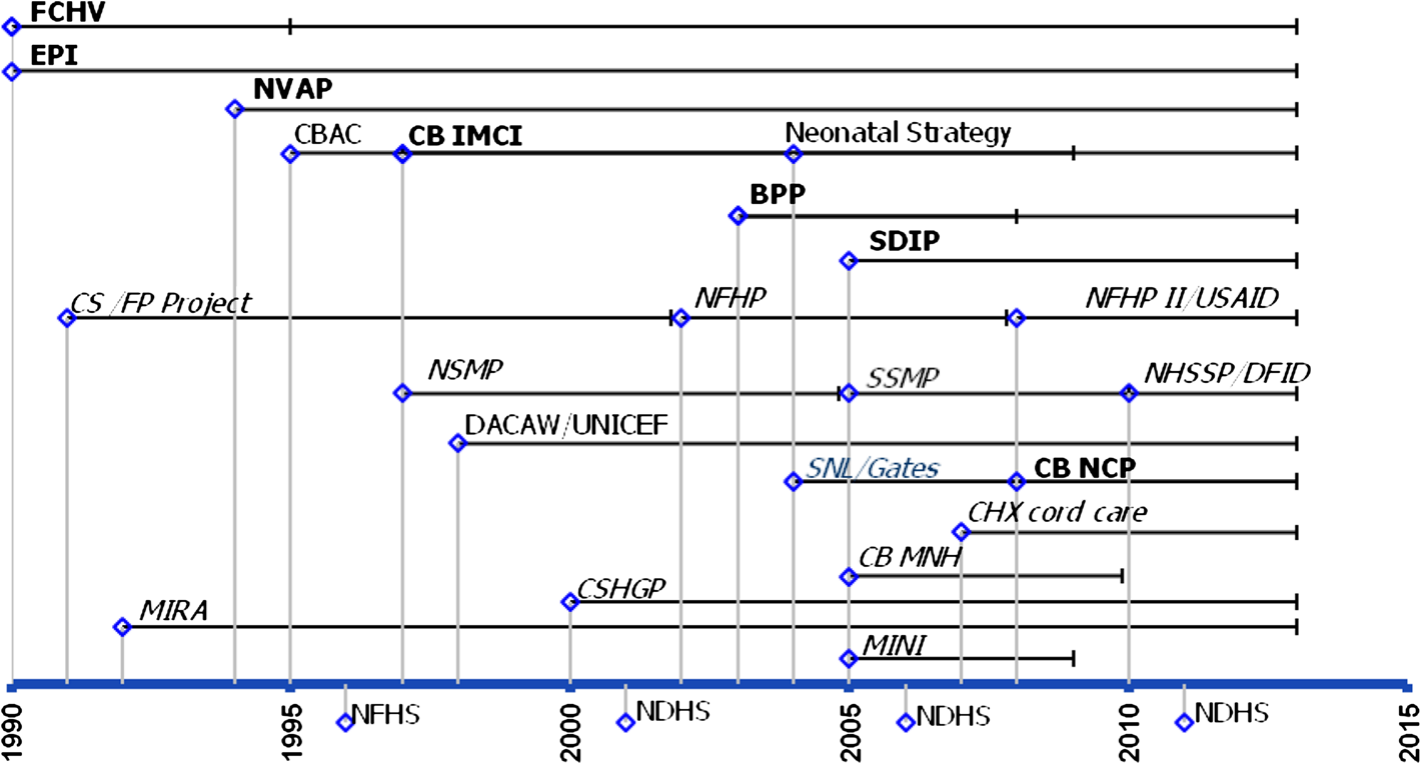
**Major maternal, neonatal and child health-related policies, programs and projects in Nepal (1990–2015).** FCHV: Female Community Health Volunteer; EPI: Expanded Program on Immunization; NVAP: National Vitamin A Program; CBAC: Community based ARI Control of Diarrheal Diseases program; CB IMCI: Community based Integrated Management of Childhood Illness; BPP: Birth Preparedness Package; SDIP: Safe Delivery Incentive Program; CS/FP Project: Child Survival and Family Planning Project; NFHP: Nepal Family Health Program; USAID: United States Agency for International Development; NSMP: Nepal Safer Motherhood Project; SSMP: Support to Safe Motherhood Program; NHSSP: Nepal Health Sector Support Program; DFID: UK Department of International Development; DACAW: Decentralized Action for Children and Women; UNICEF: United Nations Children’s Fund; SNL: Saving Newborn Lives program; CB NCP: Community based Newborn Care Package; CHX Cord Care: Chlorhexidine for Umbilical Cord Care; CB MNH: Community based Maternal Newborn Health program; CSHGP: Child Survival Health and Grant Program; MIRA: Mother and Infant Research Activity; MINI: Morang Innovative Neonatal Intervention; NDHS: Nepal Demographic and Health Survey; NFHS: Nepal Family Health Survey.

Since 1990, Nepal has developed, piloted and gradually scaled-up a broad range of facility- and community-based programs to address maternal, neonatal and child health; many of these have since been integrated with regular public health programs. Figure 2 provides a graphical overview of these programs, distinguishing between national programs (presented in bold) and sub-national programs or pilots (presented in italics) and showing support from external donors. They cover the continuum of maternal and child health and comprise integrated approaches to addressing multiple health concerns among target populations (e.g. community-based Integrated Management of Childhood Illness), highly vertical programs to address specific health conditions (e.g. National Vitamin A program) and interventions to strengthen the health system (e.g. female community health volunteers program). While neonatal health is not an explicit focus in all of these programs, they illustrate the considerable background activity prior to the introduction of focused newborn interventions, with specific components of all programs directly impacting newborn health.

Newborn survival was made a development priority for the country through a major strategic document endorsed by the Ministry of Health and Population, the Nepal Neonatal Health Strategy 2004 [28]. In an effort to develop recommendations towards the implementation of this strategy, a rapid assessment of neonatal health programs in Nepal was conducted in 2007 to analyze the status of ongoing programs and the roles of different partners, and to identify gaps and constraints in the implementation of evidence-based interventions [29]. All recent policy documents, such as the periodic health plans (9^th^ plan, 10^th^ plan and interim plan) and the Nepal Health Sector Plans I (2004–2009) and II (2010–2015) recognized neonatal health as a priority and listed it as a component of essential health care services [30-36]. Some of these policy documents also emphasize improved access for poor and vulnerable groups [33]. Taken together, these policies and strategies provide both a conducive environment and a framework for developing, piloting and implementing newborn programs in the country [37].

Table 3 provides more in-depth information for three established and two more recently initiated programs that are considered to be of critical importance in improving neonatal health based on (i) their explicit focus on delivery and the post-partum and neonatal periods, (ii) their programmatic rather than research orientation, and (iii) their aim for or ongoing national-level implementation. In principle, these were developed and implemented based on available international, regional and national evidence [38,39].

**Table 3:** Major policies and programs to improve neonatal health

**Program**	**Description**	**Newborn survival interventions**	**Evidence of program effectiveness in Nepal**	**Program timeline and scale**
**Community Based Integrated Management of Childhood Illness (CB IMCI)**	National program, which forms part of the broader WHO/UNICEF IMCI model to improve child health and survival and focuses on treatment of common childhood illness at community level through disease prevention and health promotion, in particular by improving performance of health workers, improving health services, and improving knowledge about the care of children at home and in the community.	• Early identification of newborn illness	• Increased case reporting of diarrhoea and acute respiratory infections (ARI) (0.21 and 0.16 diarrhoea episodes per child per year in areas with and without intervention respectively; 55% and 27% of all under-five children reporting with ARI in areas with and without intervention respectively) [40]	Initiated: 1997
		• Community-based management and referral of sick newborns		Nationwide: 2009
			• Decreased case severity of diarrhoea and ARI (29% and 35% of all diarrhoea cases with some dehydration in areas with and without intervention respectively; 28% and 38% of ARI cases reported as pneumonia in areas with and without intervention respectively) [41]	
			• Program scalability [42]	
			• Program contribution to overcoming problem of insufficient human resources for health [42]	
**Birth preparedness package (BPP)**	National package of interventions to encourage pregnant women, their families and communities to plan for normal pregnancies and deliveries as well as for obstetric emergencies, designed to be implemented through female community health volunteers and health workers in primary care facilities.	Education and counselling on:	• Increase in putting into practice five healthy newborn care practices ranging from 19% to 29% from baseline (42% to 71% for clean cord care, 56% to 75% for immediate wiping, 56% to 79% for immediate wrapping, 21% to 40% for immediate breastfeeding and 12% to 41% for delayed bathing) [43]	Initiated: 2003
		• Preparedness for safe delivery and promoting essential newborn care practices (clean cord, wiping, wrapping, immediate breastfeeding and delayed bathing)		Nationwide: 2008
		• Danger signs during pregnancy, delivery and the postnatal period		
		• Danger signs among newborns		
		• Tetanus toxoid vaccination		
**Community-based Newborn Care Package (CB NCP)**	A pilot program developed on the basis of CB-IMCI with a new set of interventions to improve the health and survival of newborn babies. The package reflects evolving evidence and national, regional and global experience, taking into account causes of neonatal mortality, suitability of interventions to large-scale implementation and cost. [39]	• Behavior change communication	• Ongoing assessment of the pilot in ten districts through Nepali Ministry of Health and Population with USAID, UNICEF and the Saving Newborn Lives program, and ongoing mixed-method study by Paudel et al. [40] to assess impact of the program on newborn care practices	Initiated: 2008
		• Promotion of institutional delivery and clean delivery practices at home		Ongoing: 35 districts (Dec 2012) ^1^, preparation for review and national scale-up
		• Postnatal care		
		• Community-based case management of pneumonia and severe bacterial infections		
		• Care for low birth weight newborns		
		• Prevention and management of hypothermia		
		• Recognition of asphyxia		
		• Initial stimulation and resuscitation of newborns		
**Umbilical cord care for newborns (CHX cord care)**	A pilot program currently being scaled-up, integrated with other maternal and newborn programs such as BPP and CB-NCP to prevent newborn infections and improve newborn survival by applying chlorohexidine to the umbilical cord stump.	• Use of chlorohexidine for prevention of umbilical cord infections	• 24% reduction in neonatal mortality among those who used chlorohexidine compared to those who practiced dry cord care; even greater 34% reduction among those who applied chlorohexidine within 24 hour after birth [44]	Initiated: 2007
				Ongoing: 33 districts (Dec 2012) ^2^, preparation for national scale-up
**Safe Delivery Incentives Program (SDIP) ** *also described as Maternity Incentives Program or Aama Surakchya Program*	National program to increase utilization of professional care during childbirth. It provides cash to women giving birth in a health facility and an incentive to the health provider for each delivery attended, either at home or in the facility.	• Promotion of institutional delivery and/or home delivery by skilled birth attendant	• Substantial increase (2.3% points) in probability of deliveries attended by a skilled birth attendant [45]	Initiated: 2005
		• Care for immediate newborn problems (e.g. birth asphyxia)	• No impact on neonatal mortality [45]	Nationwide: 2008

^1^Dhankuta, Morang, Palpa, Doti, Bardiya, Dang, Chitwan, Kavre, Parsa, Sunsari, Terathum, Sankhuwasava, Kailali, Myagdi, Bajhang, Banke, Kapilbastu, Arghakhachi, Mohattari, Salyan, Dailekh, Jumla, Nawalparasi, Saptari, Sarlahi, Jajarkot, Lamjung, Humla, Taplejung, Bara, Baglung, Dolpa, Rautahat, Baitadi, Rupandehi.

^2^Banke, Jumla, Bajhang, Parsa, Darchula, Baitadi, Doti, Kailali, Bardiya, Dailekh, Dolpa, Rolpa, Myagdi, Palpa, Rautahat, Mahottari, Saptari, Sankhuwasava, Morang, Sunsari, Dhankuta, Sarlahi, Nawalparasi, Kapilbastu, Arghakhachi, Humla, Kanchanpur, Baglung, Lamjung, Bara, Khotang, Taplejung, Salyan.

The community-based Integrated Management of Childhood Illness (CB IMCI) represents an established approach and is globally considered one of the best models for integrated delivery of care at family and community level for the most common illnesses (i.e. pneumonia, diarrhoea, malaria, malnutrition) during the first five years of life. The birth preparedness package (BPP) targets pregnant women, helping them and their families to be prepared for a safe delivery and for the arrival of the baby, and to recognize danger signs and seek care from a health provider when needed. It emphasizes the need to be ready for emergencies, such as blood transfusion or caesarean section, and to reduce delays in seeking care for maternal or neonatal illness (in particular newborn asphyxia, postpartum haemorrhage and severe bacterial infections). The main objective of the Safe Delivery Incentives Program (SDIP) is to increase deliveries at health institutions (i.e. hospital, primary health care center or health post and sub-health post with birthing center) and thus to provide better care for mothers and newborns during and immediately after birth. Institutional deliveries or safe home deliveries are expected to contribute to reducing all causes of neonatal mortality, in particular they can prevent newborns from dying due to birth asphyxia and severe bacterial infections. More recent programs include the community-based newborn care package (CB NCP) and the umbilical cord care for newborns (CHX cord care) program; both are in early phases of implementation and roll-out. Based on the experience of implementing these programs and results to date, these programs are being revised to integrate approaches by harmonizing efforts and messages. These changes are expected to create synergies in the delivery of better neonatal health outcomes.

As shown in Table 3 evidence of effectiveness of these programs in Nepal with respect to neonatal health outcomes, as obtained from the scientific publications and documents reviewed, is scarce. Notably, we were unable to quantify coverage of the five programs beyond a general statement about the geographical spread of implementation (i.e. national or various districts); likewise, we were unable to document equity or inequity in program implementation. To date, studies of program impact on neonatal mortality are rare. We were only able to identify two such studies, where a cluster-randomized controlled trial of a pilot showed large and statistically significant declines in cord infections as a result of applying chlorhexidine [44] and an interrupted time series study in one district failed to demonstrate any impact on neonatal mortality for the implementation of the SDIP program [45]. Current gains in reducing under-five mortality in Nepal can be plausibly linked to CB IMCI [46] but, as of yet, no evidence is available for a measurable impact on neonatal mortality. Instead or in addition to looking at neonatal mortality, several programs measured impact in term of changes in intermediate outcomes, such as birth preparedness (BPP, [43]), institutional or safe home delivery (SDIP, [45]) and case reporting and case severity (CB IMCI, [41,42]).

## Discussion

### Critical considerations across programs

Based on the data available, Nepal has made very good progress in terms of reducing child and infant mortality [3]. In order to be able to achieve further gains in child survival, the main challenge for the country will be to reduce neonatal mortality. Neonatal mortality varies greatly by wealth status and, to a lesser extent, by maternal education, caste and ethnicity and geographical location. Absolute and relative inequalities in neonatal mortality are relatively stable and interventions to date do not appear to have acted to greatly decrease or increase inequalities. As the annual rate of reduction in neonatal mortality has stagnated in recent years and as inequalities in neonatal mortality persist, labelling a country as “on track” to achieve the MDGs may divert focus and slow the momentum of ongoing efforts. Despite persistent high levels of neonatal mortality, in the last decade Nepal has witnessed a higher rate of decline in neonatal mortality (3.6 percent per annum) compared to the global average (2.1 percent per annum) and compared to progress made in neighbouring countries [47,48]. This achievement can be plausibly linked to the country’s progress with respect to family planning, antenatal and delivery care practices as well as significant improvements in infrastructure over the same period.

The Government of Nepal, together with major international donors, has implemented a broad range of programs to address maternal, neonatal and child health problems over the past twenty years. It is difficult to attribute the progress made to date to individual programs, in part because of the broad range of ongoing activities and in part because of insufficient impact data for individual programs. In particular, information on coverage and uptake of the five programs most directly concerned with reducing neonatal mortality and evidence of impact on neonatal mortality rather than intermediate outcomes is limited. Moreover, the available information is rarely disaggregated for different socio-economic groups, although descriptive data suggest that programs do not equitably reach those in greatest need [6]. Given the substantial financial and personnel resources dedicated to these programs, it will be important to carefully evaluate their future performance in terms of coverage and effectiveness.

Other programs covered by Figure 2 but not further considered in Table 3 are also likely to contribute to improved newborn health. Specific interventions, such as tetanus toxoid injections and the safe motherhood program improve coverage and quality of care of obstetric and newborn care services. Other programs are mobilizing communities and their change agents to educate mothers and their families, promoting healthy behaviors for better maternal and newborn health in different parts of the country. With respect to strengthened primary health care, female community health volunteers (FCHV) are a unique and strong cadre for all community-based programs in Nepal and play a crucial role in delivering newborn interventions under the CB NCP and CHX for cord care [49]. Beyond these front-line health workers, Nepal’s public health system is suffering from a shortage of health personnel and services, especially in rural areas. For example, many birthing facilities do not offer a 24-hour service seven days a week and, as a result, expectant mothers are often reluctant to deliver at a health facility. Therefore, greater capacity of providers at all levels and broader health system strengthening will also be necessary to improve maternal and newborn health care services further [50].

A further hidden reason behind the limited impact of neonatal health programs to date is the fact that some practices, such as immediate care for newborns and recently delivered women, are greatly affected by existing cultural norms and behaviors. In Nepal and many south-Asian cultures, birth and the postnatal period are considered ritually polluted, and the new mothers often face seclusion which undermines their ability to seek health care when needed for themselves or their babies [51,52]. Their care-seeking behavior is further limited by decision-making authorities in the household, where the ultimate decision to seek care and pay for travel, care or medication usually resides either with the male head of household or older women such as mothers-in-law [53]. As a result, new mothers might not be able to follow recommended newborn care practices due to existing family and social pressures and their inability to negotiate on these matters. Furthermore, issues related to health worker behavior, gender-friendliness in service delivery and perceived quality of care affect service utilization and compliance. In south-Asian cultures, many women do not visit health facilities as most of the providers are male, or because they have not been treated respectfully during previous visits. The design of programs and their delivery will therefore need to pay attention to implementing interventions in socio-culturally appropriate ways, in particular among socio-economically disadvantaged and hard-to-reach population groups.

The strong support from high-level policy makers, as evident in the recognition of neonatal health in the long-term health sector plan and the neonatal health strategy, has been and will be a critical ingredient of making progress. Likewise, effective partnerships between the government and a range of donors have enabled the country to develop and implement neonatal health programs and to scale these up over a relatively short period of time [37,38]. There are, however, some missed opportunities to integrate and harmonize across efforts. Some programs are implemented in a vertical fashion with distinct implementation modalities and information systems, and limited attempts are being made to harmonize messages for behavior change across programs. For example, CHX for cord care is delivered as a stand-alone program even though it clearly fits into the CB NCP and CB IMCI program framework. Similarly, the SDIP could be extended from just focusing on the number of institutional deliveries to expanding quality care for mothers and newborns. This is likely to be particularly relevant for newborn survival as the institutional delivery rate is rapidly increasing and as this early contact between health workers and mothers is an opportunity for timely diagnosis and treatment of newborn illnesses and for the promotion of essential newborn care practices. Most of these programs focus on increasing health workers’ knowledge and skills to deliver services to prevent, diagnose and manage newborn illnesses on the one hand, and on improving community behaviors and generating demand for care and services on the other hand. At the same time, efforts should be targeted more towards strengthening health systems to improve the delivery of basic supplies and equipment, such as chlorhexidine for cord care, gentamicine for newborn infections and resuscitation-kits to manage asphyxia, all of which are critically needed to prevent and manage newborn illness.

Nepal has witnessed considerable political unrest and even armed conflict during 1996 – 2006. Nevertheless the country continued to improve most of its health indicators despite some disruption to health services [54]. During the last two decades, Nepal has also undergone significant socio-economic changes, in particular improved transportation and communication infrastructure and girls’ education. International labour migration has been a recent phenomenon leading to young males not being at home to support and care for recently delivered women and their newborns but also generating greater household income due to remittance. The effect of these major societal changes on neonatal and broader health indicators among different population groups has not been yet systematically assessed.

### Strengths and limitations

We provided time trends in neonatal mortality and conducted an assessment of absolute and relative differences in neonatal mortality, based on best-available data (Table 2). There are, however, several problems with these data. Due to limited sample size, neonatal mortality was calculated for the last ten years preceding the survey, resulting in sample overlaps between estimates for different points in time. Also, the MDG baseline for 1990 and the MDG targets for 2015 in Figure 1 are estimates provided by the Ministry of Health and are thus not truly comparable to survey data for the years 1996, 2001, 2006 and 2011. Most importantly, NFHS and NDHS data are designed to be representative at the national level but may not necessarily be representative for each of our subgroup analyses, e.g. selected caste and ethnic groups; therefore, the comparison of rate differences and rate ratios and their changes across time must be made with caution.

A major limitation in relation to our review of efforts to reduce neonatal mortality is that information on relevant programs and policies and evidence of their coverage and effectiveness is based on non-systematic literature searches. Through conducting searches of the grey literature as well as the scientific literature and, in particular, through consultation with relevant stakeholders in Nepal, we are confident to have captured all important programs of direct relevance to neonatal health. We are less confident to have unearthed all available evidence on the effectiveness of different programs, especially as much unpublished additional evidence may reside with implementing organizations. Also, we did not conduct any formal quality appraisal for studies of effectiveness and have therefore not examined the evidence in terms of methodological flaws and potential bias due to non-independent evaluations.

Nevertheless, we believe that this study provides an important and much needed overview of key developments in neonatal health in Nepal over the past 20 years, capturing time trends in neonatal mortality in a disaggregated way, examining absolute and relative inequalities and providing an analysis of current experience with policies and programs.

## Conclusions

This study clearly shows that much remains to be achieved in terms of reducing neonatal mortality across different socio-economic, ethnic and geographical population groups in Nepal. In moving forward it will be important to (i) strengthen and further increase the reach of those programs that have proven to achieve good results, such as applying chlorhexidine to prevent umbilical cord infections; (ii) put in place in-depth evaluation of the effectiveness and implementation approach of those programs that are promising but whose impact on neonatal mortality has not yet been verified, in particular CB NCP and SDIP; (iii) target hard-to-reach population across the country, customizing interventions as needed to ensure that they are socio-culturally appropriate; and (iv) maximize use of available financial and personnel resources by facilitating interaction between and, where feasible, integration across neonatal health programs as well as broader efforts to promote maternal and child health.

## Competing interests

DP was employed by the United States Agency for International Development in Nepal and has been involved with the management of neonatal health programs.

## Authors’ contributions

DP and ER had the original idea for this paper. DP carried out searches and data analysis, and prepared the first draft. ER, IBS, MS advised on methods and interpretation of findings. ER, IBS, MS reviewed and revised the draft manuscript. All authors read and approved the final manuscript.

## Pre-publication history

The pre-publication history for this paper can be accessed here:

http://www.biomedcentral.com/1471-2458/13/1239/prepub
